# Magnetowetting of Ferrofluidic Thin Liquid Films

**DOI:** 10.1038/srep44738

**Published:** 2017-03-17

**Authors:** Srinivas Tenneti, Sri Ganesh Subramanian, Monojit Chakraborty, Gaurav Soni, Sunando DasGupta

**Affiliations:** 1 Department of Chemical Engineering, Indian Institute of Technology, Kharagpur 721302, India

## Abstract

An extended meniscus of a ferrofluid solution on a silicon surface is subjected to axisymmetric, non-uniform magnetic field resulting in significant forward movement of the thin liquid film. Image analyzing interferometry is used for accurate measurement of the film thickness profile, which in turn, is used to determine the instantaneous slope and the curvature of the moving film. The recorded video, depicting the motion of the film in the Lagrangian frame of reference, is analyzed frame by frame, eliciting accurate information about the velocity and acceleration of the film at any instant of time. The application of the magnetic field has resulted in unique changes of the film profile in terms of significant non-uniform increase in the local film curvature. This was further analyzed by developing a model, taking into account the effect of changes in the magnetic and shape-dependent interfacial force fields.

Process intensification in the micro-scale domain has been garnering attention due to their inherent abilities and advantages to control the transport phenomena with a minimal expense of resources^1^,^2^. Micro-Electro-Mechanical-Systems (MEMS) and nanotechnology have been paving way for newer and more advanced discoveries^3^ such as Polymerase Chain Reaction (PCR)^4^, microsystems for DNA amplification and identification^5^, enzyme linked immunosorbent assay (ELISA)^6^, capillary electrophoresis^7^, electroporation^8^, biochips for the detection of hazardous chemical and biological agents^9^ and microsystems for high throughput drug screening and selection^10^,^11^. Another well defined and established application of microfluidics and microfabrication is in the domain of device fabrications, utilizing photolithography^12^,^13^. Integrated circuits (IC’s), the basic building blocks of any electronic device are becoming more complex, efficient and affordable due to the advancements in photolithography. However, the performance of IC’s is limited by their significant heat generation, creating a need for the rapid development in alternate cooling strategies, such as micro-heat pipes^14^, thin film micro- refrigerators, etc^15^,^16^. Droplet based micro fluidics^17^ that operate on electrowetting is also becoming a popular alternative for micro-chip cooling.

The theory of electrowetting^18^,^19^ along with its applications have been reviewed and reported by various researchers. The phenomenon of droplet electrowetting has been exploited on various surfaces and processes, such as paper^20^, carbon nanotubes^21^, liquid infused films^22^ and more recently for energy harvesting and storage^23^ and hot spot cooling enhancement^24^,^25^. Droplet motions with specific applications in lab on a chip devices have been extensively analyzed and reported by various researchers^26^,^27^,^28^,^29^. Dynamics of droplets subjected to various modes of perturbation such as acoustic^30^, thermal^31^, optical^32^ and magnetic^33^ have been studied extensively, whereas droplet manipulation through magnetic actuation, with advancements in point of care medical diagnostics has been reported by Shin *et al*.^34^.

The related concepts have been further extended to thin liquid films as well. Subjecting a thin liquid film to external perturbations resulting in the actuation of liquid motion is used to probe and manipulate the transport phenomena near the contact line region. The analysis of the dynamics of partially wetting thin liquid films, experiencing an external perturbation, is a complex process^35^,^36^,^37^. Evaporating thin liquid films may provide very high heat transfer coefficients across the liquid-vapour interface^38^,^39^. The physics associated with the dynamics of thin liquid films undergoing cycles of evaporation and condensation was first studied and quantified by Potash and Wayner^39^. The hydrodynamic flows associated with thin liquid films when subjected to continuous evaporative-condensing cycles in micro heat pipes has been studied and reported by Swanson *et al*.^40^. Since then, the response of thin liquid films subjected to various modes of perturbation such as thermal^38^,^41^ and electrical^35^ have been investigated and reported. Extensive reviews on intermolecular forces and interactions present in a thin liquid-solid-vapour system have been presented by Derjaguin *et al*.^42^. The contact angle hysteresis and equilibrium dynamics of thin liquid films have been reviewed and reported by Starov^43^, whereas the wetting and spreading near the three phase contact line has been investigated by Bonn *et al*.^44^. Argade *et al*.^41^ probed the dynamics of evaporating and condensing thin liquid films subjected to thermal perturbations. Bhaumik *et al*.^35^ studied the contact line dynamics of thin liquid films and reported the contact line instabilities associated with satellite drop formation, subjected to electrical actuation. Recently, Chakraborty *et al*.^45^ investigated the effect of introducing negatively charged nano particles to the thin liquid film (TLF) and studied the associated dynamics by subjecting the thin film to DC electric field. They observed that the addition of nano particles not only enhanced the thermal conductivity of the motive liquid but also increased the net forward motion of the liquid meniscus. These studies on the dynamics of thin liquid films underscore the effectiveness and ease of applying electrical mode of actuation to the thin liquid film and the potentiality of direct application to microscale processes.

However, the problem associated with electrical actuation is the contact line pinning closely related to the homogeneity and smoothness of the substrate, as well as the localized nature of Maxwell stress, prevalent only near the contact line. The other difficulty in electrowetting is that the source (of energy) has to be in direct contact with the medium, thereby posing an operational problem as in biological applications. The application of magnetic field to droplets and studying their dynamics^33^,^46^ have shed light on the alternative options available for fluid manipulation and corresponding applications.

Any particular mode of perturbation would result in a change of the surface energy of the system, thereby resulting in the TLF equilibrating to a new configuration, involving both, transport of and within the film. Precise and optimal control of the parameters would help in a myriad of applications in microflows. Despite the extensive reports on the detailed dynamic and transient studies for thin liquid films, it was observed that, there was a dearth of studies, pertaining to magnetic actuation. It was anticipated that the advantage of influencing the film through magnetic actuation would increase the versatility of application, because unlike electrical actuation, the magnet need not be in physical contact with the system. Also, since the magnetic force is a body force it will act on the entire film, unlike the electrical component of the Maxwell stress tensor which acts solely at the contact line. Additionally, it is possible to partially circumvent the need for the solid surface to be very smooth and devoid of surface imperfections. Hence, it would be more insightful to delve deeper into the dynamics of thin liquid films being subjected to a magnetic field and characterize them using a non- intrusive method, which is the motivation for the present study.

## Results

### Characterization of the Magnetic Force Field

The net differential magnetic force experienced at any point in the permeable medium is obtained from the general Maxwell stress equation^47^ in the tensorial notation *(denoted in bold-face)* as in,


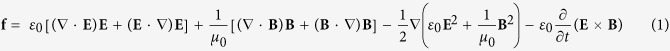


where,

**E** = Electric field

**B** = Magnetic field

**H** = Magnetic field strength

ε_0_ = Permittivity of free space (vacuum)

μ_0_ = Permeability of vacuum

(r, *θ*, z) = Coordinates representing the magnet used in the present work

t = time

**f** = Overall electromagnetic force due to the applied field (both electric and magnetic)

The three cylindrical coordinates that have been chosen to represent the magnet are (r, *θ*, z) respectively. Since the magnet is θ-symmetric, only the two dominating coordinates vis-à-vis the (r, z) coordinates are considered for the present formulation. The first term indicates the effect of the electric field and its gradient, whereas the second term indicates the magnetic field and its gradient. The third and the fourth terms are a result of electrically induced magnetic field and magnetically induced electrical field^47^. The effect of the gradient in magnetic field is nullified in agreement with Gauss’ law and hence, the net force per unit volume, due to the magnetic field alone, at any given point can be written as,


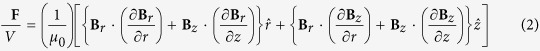


where,


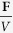
 = Force per unit volume along radial and axial directions due to the applied magnetic field

**B**_*r*_ = Radial component of the magnetic field

**B**_*z*_ = Axial component of the magnetic field


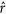
 = Unit vector along the radial direction


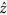
 = Unit vector along the axial direction

The relative magnetic permeability of the EFH series-1 ferrofluid is 2.6 and the corresponding susceptibility is 1.6 (as obtained from the material safety data sheet (MSDS)^48^, Ferrotec™). In the present case, the ferrofluid is diluted with n-Heptane such that the concentration of the resulting solution is 10% by volume of ferrofluid. The magnetic susceptibility is observed to have a direct relationship with particle concentration as reported by Ivanov *et al*.^49^ and is calculated to be 1.16 for the present case and is assumed to be constant throughout the series of experiments.

### Simulation of the magnetic field

**Figure 1:**
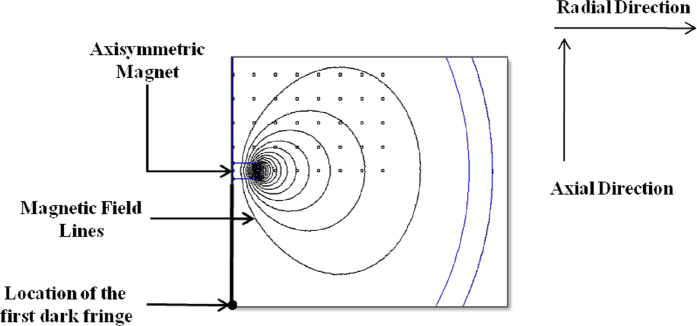
Schematic of the simulation of the Net Magnetic Field (as obtained from the FEMM simulation).

The magnetic field is simulated by considering the axisymmetric problem in the FEMM (series 4.2) with dimensions and the material being exactly the same as that of the magnet used in the present work. The entire domain is discretized into 161509 nodes. A schematic of the simulation framework is presented in Fig. 1. The magnetic field was simulated by considering a rectangular mesh grid of size (50 μm × 50 μm) to obtain the accurate strength and orientation of the magnetic field. The spatial domain was marked with nodal points each separated by 1 cm in both r and z directions. This was done to increase the feasibility and ease of plotting the magnetic-field profiles from one nodal point to the other in the aforementioned direction. Each pair of nodal points in any direction are separated by 199 data points containing the information regarding field strength and orientation.

The net magnetic force is calculated in the domain having the limits of r (0 to 4) cm and z (3–5) cm in the radial and axial direction respectively. This domain is discretized into points in such a way that the separation in both radial and axial direction is 50 microns. At each of these points, the effective radial and axial force components are calculated by simplifying equation (2) to equation (3). The axial component acts perpendicular to the surface of the silicon wafer and points towards the wafer. This component gets nullified by the normal reaction force from the surface of the silicon wafer. The radial component of the magnetic field plays a critical role in the movement of the meniscus. The total radial magnetic force could be given as


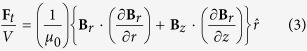


where,


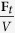
 = Net force per unit volume along radial direction

**Figure 2:**
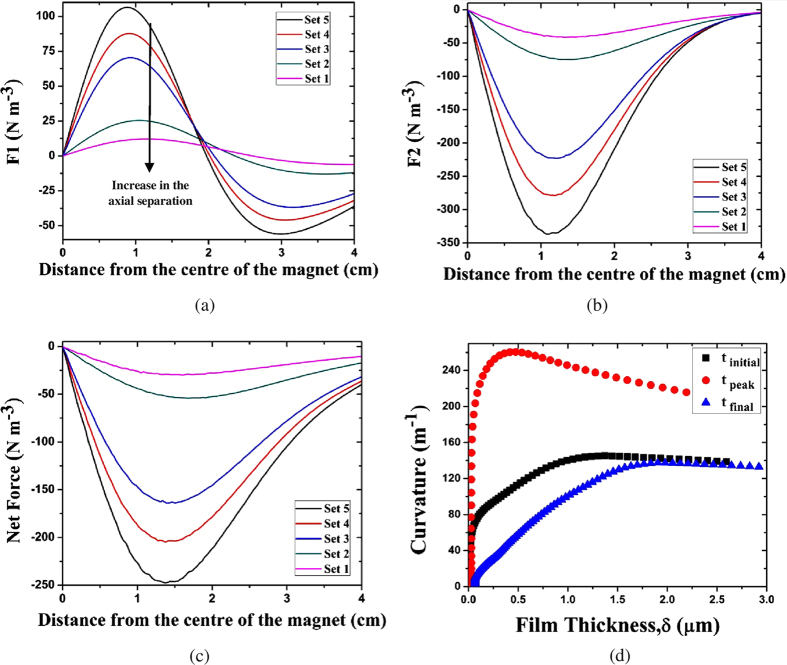
Variation of magnetic force with radial distance for different vertical position of the system from the disk magnet. The legends are representative of the axial positions (vis-à-vis field intensities); (**a**) Force 1 (F1) (**b**) Force 2 (F2) (**c**) Net Force. The magnitude of the net magnetic force decreases in accordance with the results reported from Set 1 to Set 5, as depicted in Table 1. (**d**) Variation of the thin film curvature as a function of film thickness at three instants of time upon application of magnetic field (Set 3).

For brevity, the first term on the right hand side of equation (3) is denoted as Force 1 (F1) and the second term as Force 2 (F2). These two terms are representative of the effective radial components of the net force, owing to the applied magnetic field. The values of the radial component of external magnetic field, **H**, at every point is obtained from the computed FEMM simulation which is used to calculate the induced magnetic field using the relation **B** = μ**H**, considering μ = 1.16μ_o_. The induced magnetic field data is subjected to polynomial curve fitting of suitable order and the corresponding point derivatives are obtained. These values are substituted in the above equation, yielding F1 and F2. Figure 2 (a through c) shows the variation of F1, F2 and the net force as a function of the radial distance (r) ranging [0, 4] cm from the centre of the magnet at various axial positions, as depicted in Table 1.

The decrease in the magnetic field intensity with an increase in the axial distance of the magnet from the system, results in a decrease in magnitude of the forces F1 and F2 as depicted in Fig. 2(a,b).

It is to be noted that the field lines are oriented in the 3D space and a progress towards the centre of the magnet would appear as though the field lines are finely oriented along the z-direction, but a progress away from the centre of the magnet, would make it evident that the field lines diverge along the radial direction. The resulting orientation of the magnetic field lines is the reason for the differential evolution in the magnitudes and directions of the forces, F1 and F2. In accordance with equation (3), it could be infererd that, the term corresponding to F1 is dependent on both **B**_r_ and 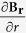 and the magnitude of **B**_r_ is low when compared to **B**_z_ due to the aforementioned orientation of the field lines. The change in the direction of F1 could be explained by the fact that, along the radial direction, the magnitude of **B**_r_ initially increases and so does the rate of change in **B**_r_ along the radial direction 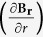, as **B**_r_ continues to increase, the rate of change decreases and ultimately becomes zero, when **B**_r_ attains a maximum value. After attaining the maximum value although **B**_r_ is positive, the rate of change becomes negative resulting in a change in the direction of the force curve. Since the point at which **B**_r_ attains a maximum value, shifts closer to the centre, with a decrease in the axial distance, the value at which the force F1 becomes zero also decreases, as depicted in Fig. 2(a).

Since the maginitude of **B**_r_ decreases with an increase in the axial position of the magnet from the system, it would be logical to deduce that the magnitude of 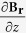
would also decrease and hence the force F2 is always negative in the present scenario.

It is to be noted that the geometric centre of the magnet (Fig. 2(c)) does not coincide with the magnetic centre for the magnet used herein. The negative value of radial force component signifies that the resultant magnetic (field) force is along the direction opposite to that of the r vector and thus the liquid flows towards the centre of magnet and drives the film forward. The axial distance of the system from the surface of the magnet is varied for each set of experiment as shown in Table 1.

It should be pointed out here that the present study is restricted to symmetrical fields without considering possible effects of other parameters such as varying the field orientations, particle size, particle concentrations etc. It is however imperative that the dynamics would be both quantitatively and qualitatively different and would be the subject of a future study.

## Discussion

The resulting effect on the extended thin ferrofluid film under the influence of magnetic field is observed in the form of significant advancement of the film. The dynamic changes in the flow parameters such as velocity and acceleration as well as the slope and curvature of the moving meniscus are evaluated. The extended thin liquid film could be broadly classified into three regions based on the type of molecular interactions and their associated significance on TLF^39^,^50^. These regions are termed as adsorbed thin film region wherein the van der Waals’ force dominates, capillary region wherein the surface tension force dominates and an interim transition region that acts as a bridge between these two regions. Although the transition is smooth, these regions are primarily identified by their relative thicknesses and therefore indirectly by the magnitude of the forces acting on the individual regions.

Since the entire film consists of the pre-cursor layer, it would be logical to assume that the van der Waals’ force should be taken into account across all the three regions, but the magnitude of the disjoining pressure scales with the film thickness as: B/δ^4^ where, δ is the film thickness and B is the dispersion constant^42^,^51^,^52^,^53^.

The relative thickness of the capillary region is typically in the range of 1 μm and above, whereas that of the adsorbed thin film is about 1–50 nm. Hence the magnitudes of these forces would be very different in case of the adsorbed thin film and the capillary region. The classification has been widely used in the existing literature^42^,^54^,^55^ and is followed herein. The transient dynamics observed are not merely caused by the magnetic field but also by the spatial orientation of the magnet as well as other implicit effects which are a consequence of the applied magnetic field. These effects would be discussed in the subsequent sections.

The effect of inter-particle interaction has been neglected in the present case owing to the fact that the size of the particles and the magnitude of the applied magnetic field are in the acceptable range as reported by various researchers^56^,^57^,^58^. It is anticipated that any change in the properties of the ferrofluid (as a result of changing the type, concentration, domain orientation etc.) would significantly affect the response of the liquid meniscus^56^,^59^.

### Dynamics of the Thin Film

Post application of the aforementioned magnetic field, it has been observed that the fringes that are located close to the junction of the adsorbed thin film and the transition region i.e. in the vicinity of the contact line recede, albeit for a very short period of time (about 2–3 s), whereas those close to the capillary region, advance. This results in a sudden rise in the curvature near the contact line as shown in Fig. 2(d).

This phenomenon, including its duration, is strongly dependent on the intensity of the applied magnetic field. The time to attain the peak curvature is termed as peak time ‘t_peak_’ and the corresponding film properties are discussed in the subsequent sections. The thickness of the adsorbed thin film is observed to be decreasing up to the peak time with a significant and sudden advancement in the capillary region. After t_peak_, the entire film begins to advance and a small increase in the adsorbed thin film thickness is observed. Table 2, shows the variation in adsorbed thin film thickness at three instants of time, initial, peak and final, for various sets of magnetic field intensities. For the experiments reported herein, t_peak_ is about 2–3 seconds, whereas the time needed for the meniscus to attain an equilibrium shape once subjected to a fixed magnetic field is about 35 seconds.

The present results are also invariant under a field inversion in the axial direction. When the magnitude of the applied magnetic field was reduced gradually, the net displacement of the moving meniscus was found to decrease. These results are indicative of the fact that the dynamics of the thin liquid film is invariant under the condition of field inversion.

The experimental setup has been strategically placed in such a way, so as to coincide the first dark fringe with the centre of the magnet. The task of correctly coinciding the centre of magnet with the first dark fringe is accomplished by firstly marking the position of the permanent magnet, in the absence of the film. Once the coordinates are marked and fixed, the magnet is moved and placed at a location far from the experimental system wherein its effect would not be felt. Successful adjustment of the position of the permanent magnet is accomplished through the use of LEICA™-FELM platform. The magnetowetting setup, which would be discussed subsequently, is now placed below the microscope and the liquid is injected into the system, forming an extended meniscus and associated interferometric fringes. Thereafter, the position of the first dark fringe is precisely adjusted such that it exactly coincides with the pre-marked coordinates that represent the centre of the magnet. Once the first dark fringe is placed at the required coordinates, the magnet is brought back to its pre-fixed position. It is to be noted that initially a part of the thin film with a thickness less than 100 nm, comprising of the adsorbed thin film and part of the transition region remained on the right side of the magnetic centre, while the rest of the film (capillary and the remaining part of the transition region) were on the left of the magnetic centre. It is evident from the net force plot (Fig. 2(c)), that the maximum magnetic force is close to the centre of the magnet. It can be hypothesized that, any differential element of the liquid in the film nearer to the centre of the magnet (in this case, the portion of the film towards the adsorbed thin film region) will experience a relatively less tangential magnetic pull in comparison to the portion of the film farther from the centre of the magnet. This is due to the fact that, though the magnetic centre coincides with the first dark fringe, the expanse to the right of the magnet corresponds to the adsorbed thin film region and a part of the transition region whereas that to the left corresponds to the capillary region and the remaining part of the transition region.

The effect of the magnetic force, being a body force, inherently depends on the volume of the liquid element on which it acts (force would be greater for the thicker part of the film). In this case, the volume of the liquid to the right side of the magnetic centre (adsorbed thin film region and part of the transition region) is relatively less in comparison to the volume of the liquid to the left (capillary region and the remaining part of the transition region). Hence, the entire volume of the liquid moves towards the centre of the magnet from both the ends, resulting in a rise in curvature for a brief period of time, near the contact line region (thickness ≈ 100 nm). However, within a short period of time (about 2–3 s), the enhanced flow from the capillary region outweighs that from the ultrathin film region, creating a net motion of the entire film in the forward direction (towards the right side of the magnetic centre). The liquid element of the film farther from the centre would advance with more acceleration in comparison to the element which is relatively nearer to the centre of the magnet following the net magnetic force as depicted in Fig. 2(c).

The second contribution to the curvature rise could be explained by the axisymmetric orientation of the field lines. Each differential element of the film moves radially inwards towards the centre of magnet. So each element has two components of velocity in the plane on which the film rests; one in the x direction and the other transversal to the x direction. The y-component of momentum applies a net force on the control volume resulting in the deformation of the film and in the present case, also causing a sudden rise in curvature.

**Figure 3:**
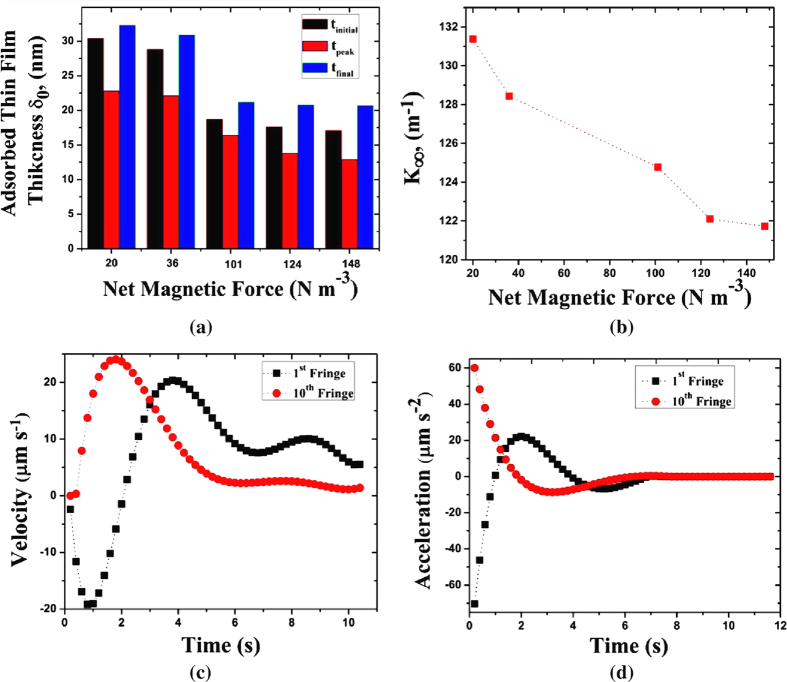
Variation in film properties with net magnetic force (**a**) adsorbed thin film thickness (**b**) curvature of thin liquid film at the capillary end. Dynamic change in thin film (**c**) Velocity and (**d**) Acceleration.

It could be inferred from the augmented Young Laplace equation^35^,^45^,^50^,^60^, that the rise in the curvature of the capillary region applies a net capillary suction causing the film to retract. The suction is predominantly felt by the thinner part of the film (first few fringes) wherein, the relative magnitude of the forward acceleration is lower in comparison to those in the vicinity of the capillary region. This continues up to a specific instance of time leading to the peak curvature. After the peak point, the entire film is observed to be advancing with an increase in acceleration, followed by a fall in acceleration (the dynamics of which are elucidated subsequently) and finally coming to a rest at the new equilibrium position. The temporal variation in the adsorbed thin film thickness and the curvature in the capillary region (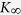) as a function of the variation in the net magnetic force, as presented in Fig. 3(a) and (b), are consistent with the simulation results depicted in Fig. 2. Once the portion of the film crosses the centre of the magnet, it starts to experience a net magnetic pull in the negative x direction resulting in deceleration and the eventual equilibration of the film to a new position.

The results depicted in Fig. 3(a) show the temporal variation in the adsorbed thin film thickness with variation in the net magnetic force. Although the net magnetic force is varied, the variation in the adsorbed- thin-film thickness is not large, suggestive of the fact that the film merely translates and causes a minor change in the thickness across its length. Although the film thickness at *t*_*final*_ appears to be saturated, the values indicated are within the range of error magnitude and hence could be safely neglected.

However, the effect of changes in the property of the ferrofluid could influence the dynamics of the adsorbed thin film, due to the fact that, lower is the particle size, greater would be the susceptibility of the particle to be adsorbed on the surface due to the combined effect of van der Waals’ force and the applied magnetic field thereby resulting in a net increase in the adsorbed-thin-film thickness with the applied magnetic field. The observations are indicative of the fact that, not only the intensity of the magnetic field but also the orientation of the field lines explicitly contribute to the dynamics of the thin liquid film and hence causality dictates that the film dynamics are field specific. Although there appears to be no experimental evidence, it would indeed be an intriguing conjecture.

### Velocity and Acceleration

The trends in velocity of the first and the tenth fringe from the contact line are depicted in Fig. 3(c). The first dark fringe, corresponding to a thickness of about 0.1 μm, is observed to be receding initially, up to about 3 s in the negative x direction. This could be explained by the dominance of capillary suction, with an initial increase in the values of the local curvature over the magnetic force in the region. However, as the fringe recedes further; the magnetic force field starts to become significant as compared to the capillary suction and this portion of the film, reverses its direction of motion and starts to move in the positive x direction, with an acceleration, until it reaches the magnetic centre. From this point onwards, the magnetic force tends to drive it in the opposite direction, with the rest of the film.

However, the thicker part of the curved meniscus, represented by the 10^th^ dark fringe shows an increase in velocity and a decrease in acceleration right from the beginning and eventually equilibrating to a constant velocity. Right from the initial instance of time, the 10^th^ dark fringe is in the region where the magnetic force predominates over the capillary suction and thus the film in this region advances. It starts to advance up to the same point wherein the remaining part of the initial film crosses the magnetic centre, as discussed earlier. Figure 3(d) shows the trend in acceleration of the first and tenth dark fringes subjected to magnetic field at Set 3.

### Theoretical Model

**Figure 4:**
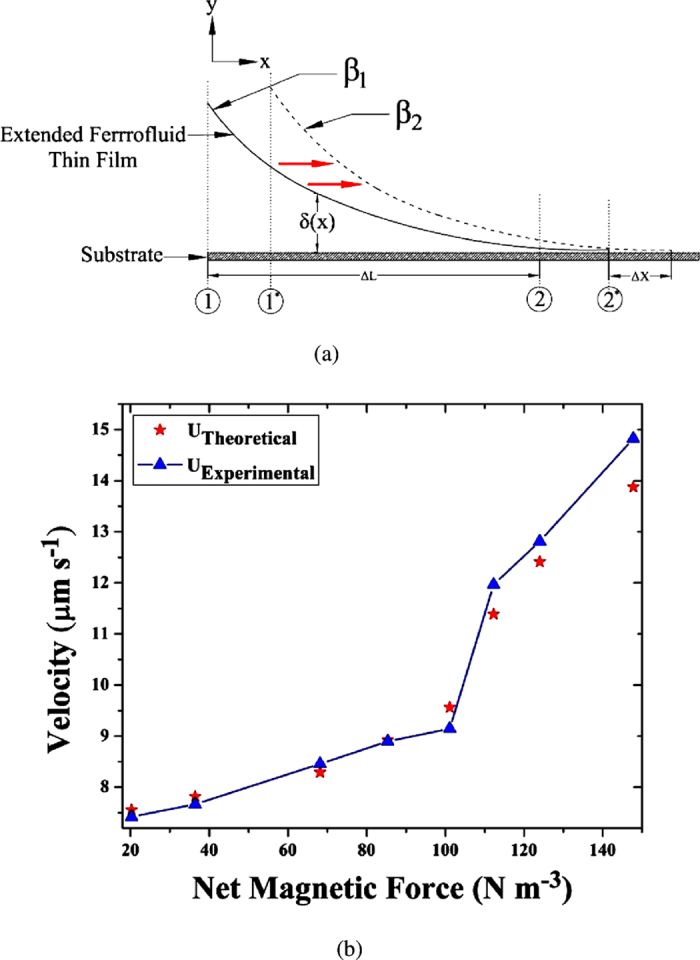
(**a**) Schematic of the advancing extended thin ferrofluid film in presence of magnetic field. The locations 2 and 2^*^, represent the locations of the first dark fringe, on the application of different magnetic forces, β_1_ and β_2_ where β_2_ > β_1_; (**b**) Comparison between the theoretical and experimental velocity of the TLF with different magnetic field intensities. The solid line is only a guide to the reader’s eyes.

The experimental evidence is imperative of the fact that the net advancement of the film increases with an increase in the magnetic field intensity. This seemingly intuitive fact is found to take place due to the nature of the TLF in response to the applied magnetic field. The rise in curvature associated with a small increase in the thickness of the adsorbed thin film is indicative of the net advancement of the TLF. The net advancement could be quantified by the physically measurable parameters, such as the average velocity of the TLF. The difference in the net pressure inside the film at both ends, namely the capillary and the adsorbed thin film region (Fig. 4(a)), creates the flow of liquid towards the contact line. The net displacement of the film subject to a particular magnetic force is represented in Table 3, wherein it is apparent that the net displacement (the difference between the equilibrium locations of the meniscus pre and post application of the magnetic field) of the thin film increases appreciably with increase in the net magnetic force.

To model the dynamics of the corresponding liquid flow, a control volume approach is used. The governing equation for liquid flow in a thin film has been extensively studied by Wayner *et al*.^39^,^50^. Using the lubrication approximation^35^,^45^,^60^,^61^, the governing equation for liquid flow inside a thin film could be modified so as to include the body force (magnetic force) term and is given as


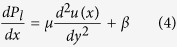


where, μ denotes the viscosity of the liquid and *β* denotes the net magnetic force per unit volume. The value of *β* at a given axial position is obtained from Fig. 2(c), by considering a line average along the radial direction.

Integrating the above equation with the appropriate boundary conditions (no slip at the wall and no shear at the interface), the average velocity is obtained as


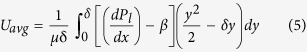



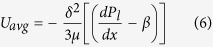


where, δ represents the film thickness at a specific location (along the x-direction) inside the control volume of a finite length (*∆L*) (Fig. 4(a)). The control volume in the present case represents the expanse between the two regions of the extended meniscus, one region with an almost constant curvature *K*_*∞*_ (the capillary region, point 1 in Fig. 4(a)) and the other region, close to the contact line (point 2 in Fig. 4(a)). Hence, the modified expression for the average velocity could be represented as


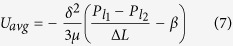


The pressure jump across the liquid-vapor interface at any given instance of time can be approximated at the two ends of the film as


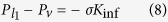



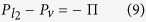


where, ∏ denotes the retarded form of the disjoining pressure in the transition region and can be expressed as 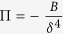, where B^42^,^50^,^53^ is the retarded dispersion constant.

Accordingly, the pressure difference along the control volume is expressed as,


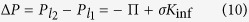


Substituting equation (10) in equation (7), yields


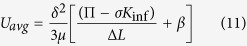


The *in-situ* evaluation of the dispersion constant is performed at the start of the experiment, following an established methodology^41^,^42^,^45^,^60^ and found to be −6 × 10^−30^ N m^2^. Taking an average value of the film thickness for the control volume, the contact line velocities are obtained for all the experimental cases experiencing different net magnetic forces. A reasonable agreement between the velocity calculated using equation (11) with the experimentally obtained one (error 4–8%) is observed and presented in Fig. 4(b).

It may be worthwhile to probe the region with more experimental points wherein the net magnetic force is between 100–150 N m^−3^ (due to the significant change in the variation of the velocity with force, as depicted in Fig. 4(b)). However, we note that the values of the net magnetic force are controlled by precisely varying the position of the magnet and the least count of this positional variance is about 0.1 cm. The data points in Fig. 4(b) encompass all the possible positions of the magnet resulting in variations in the magnitude of the applied magnetic force

## Methods

### Experimental Setup and Materials Used

**Figure 5:**
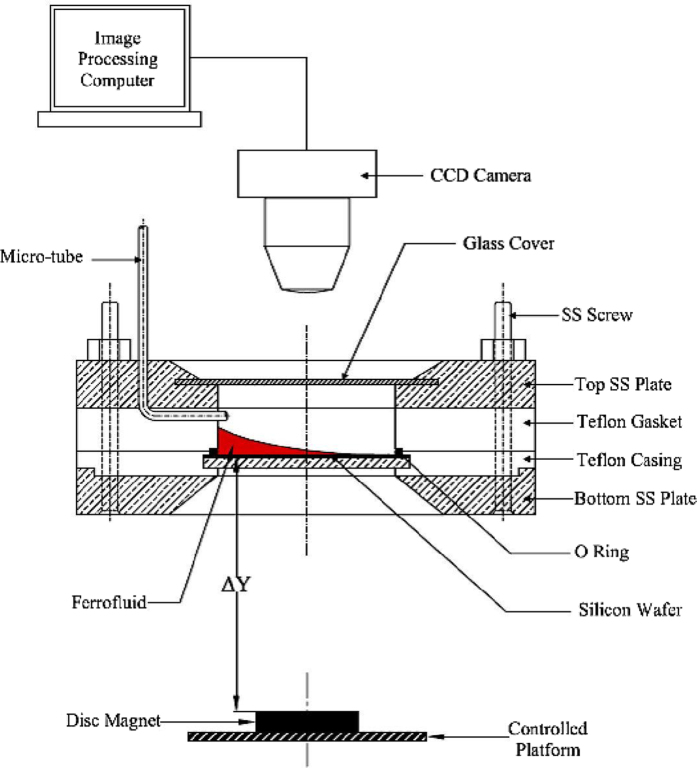
Schematic of the experimental setup.

The experimental setup primarily consists of a closed cell (Fig. 5) enclosing a thoroughly cleaned silicon wafer, with an extended meniscus of ferrofluid (EFH series-1 ferrofluid manufactured by Ferrotec™) and vapour space above the liquid. The main purpose of closed cell is to isolate the system and minimize the influence of external perturbations and impurities, including dust particles from the surroundings, which otherwise would adversely affect the experiments. The curved liquid meniscus was continuously monitored using a CCD camera (LEICA™ DFC450) mounted on a microscope (LEICA™ DM-LM) using 10X and 20X objectives. For the 10X and 20X magnifications, each pixel represents an equivalent diameter of 0.486 μm and 0.243 μm respectively.

The system consists of a uniform flat base, made of stainless steel on which the cleaned silicon wafer rests. The base plate was covered with a Teflon gasket that has an inlet port carved on it for ease of liquid injection. A Nitrile O-ring was strategically placed between the base and the Teflon gasket, so as to make the system absolutely leak proof (Fig. 5). The system was then covered with a stainless steel casing similar to that of the base, but with a transparent glass window, through which the film could be viewed and monitored by the camera. The whole setup was tightly bolted to seal the chamber, thereby preventing leakage and evaporative losses.

The silicon wafer that was used in all the experiments possesses an inherently high surface energy, thereby making it highly vulnerable to contamination. Hence, stringent protocols were followed at the start of the experiment. Every component of the experimental cell was left overnight in pure ethanol, for effective cleaning. The silicon wafer was subjected to Piranha cleaning by dipping it in a reactive mixture of 30% H_2_O_2_ with H_2_SO_4_ in the proportion of 1:3 (v/v), for the removal of organic contaminants from the silicon surface. The wafer was then thoroughly rinsed with DI water to remove traces of the Piranha solution. The setup was assembled in a class 100 Laminar Flow hood (MFD-V-W-2400, Micro Flow Devices India Private Limited) to ensure that the internal chamber of the assembled cell was free from dust or foreign particles.

EFH series-1 ferrofluid manufactured by Ferrotec™ was initially considered as the liquid medium to study the film dynamics. However, it was found to be highly viscous and was diluted with n-Heptane (the initial particle concentration of the as-obtained ferrofluid was 8% by volume) such that the solution comprised of 10% by volume of the chosen ferrofluid. The two liquids were completely miscible and the resulting solution with reduced viscosity and with suspended nanoparticles was observed to be retaining its ferrofluidic property (the liquid was found to move when subjected to a magnetic field). The viscosity of the resultant ferrofluid solution (FFS) was measured to be 3.76 × 10^−4^ (Pa.s), using a rheometer (Kinexus pro+, Malvern^®^). This solution, with a magnetic nano particle concentration of 0.8% (v/v) was used for the entire set of experiments. The surface tension of the solution was measured using pendant drop technique using a goniometer (290-G1 Ramehart™, Germany) and was found to be 20 mN/m. A commercial grade Ceramic-5 magnet having a surface external field intensity of 3950 Gauss, in radial direction was used as the source of magnetic field.

**Figure 6:**
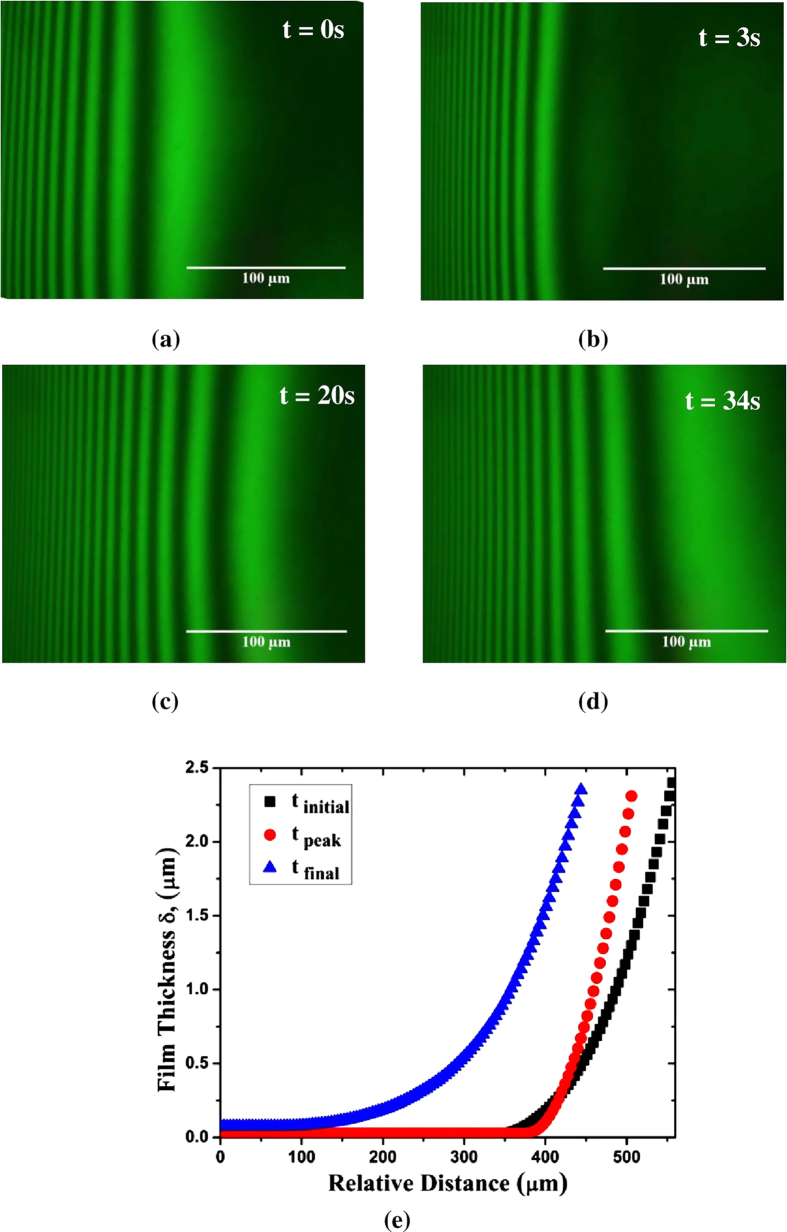
Image sequence depicting the dynamics of the thin film when subjected to a magnetic field from time t = 0 s to t = 34 s. The initial and final images are indicative of the initial and final equilibrium positions of the film, before and after application of the magnetic field for Set 3. (**e**) Spatio-temporal variation in film thickness for magnetic field of Set 3.

The FFS was introduced through a capillary tube, embedded into the Teflon gasket using a 2 ml surgical syringe. The experimental setup as a whole was tilted by an angle of 10^0^ with the horizontal and an extended meniscus was readily formed. Sufficient time was allowed for the equilibration of the system. The film was then subjected to an axisymmetric non-uniform magnetic field by positioning the permanent magnet with aforementioned specifications, in such a way that the first dark fringe, with an approximate thickness of 100 nm, coincided with the centre of the magnet. Successful positioning could be accomplished by the use of LEICA™ FELM platform. The time taken for exact positioning of the magnet at the required location was of the order of 0.1 seconds which was significantly less than the response time of the film (the time taken for the movement of the film was close to 1 second). Monochromatic light of wavelength (λ = 543.5 nm) was used as the light source. The film was continuously monitored via a microscope (LEICA™ DM-LM) as naturally occurring interference fringes, as shown in Fig. 6 (a through d) were readily visible and were analyzed using image analysing interferometry to characterize the shape of the moving meniscus. This *in-situ*, non-obtrusive technique was extensively studied and reported in literature^35^,^38^,^39^,^41^,^50^,^55^,^62^.

### Image Analysis

The entire sequence of the movement of the extended meniscus was recorded in the form of a movie clip, using a CCD camera (LEICA™ DFC4500). Images were extracted from the captured videos using Xilisoft HD video converter v.5.1 with a time interval of 0.2 s. The image resolutions obtained were 1920 × 2560 (pixels) and 960 × 1280 (pixels) for 10X and 20X magnifications respectively. The profile of the light intensity of each pixel, along a line perpendicular to the fringes, was expressed in terms of their gray values having the intensities in the range [0, 255] using ImageJ ^®^ v. 2. The temporal variation in the relative gray value of each pixel, at any position in the line profile was calculated and the thickness profile of the thin liquid film was obtained^36^,^48^ and presented in Fig. 6(e) for a fixed strength of magnetic field (Set 3).

### Evaluation of the velocity and acceleration of the moving meniscus

The captured videos corresponding to the film dynamics in response to the applied magnetic field were extracted into an image sequence depending on the time lapse as mentioned earlier. A sample of the extracted images (for Set 3) is presented in Fig. 6 (a through d). In each image, the positions of the dark fringes were located and compared with their corresponding positions in the subsequent image. This process was repeated for the whole length of the experimentation time. After obtaining the relative distance travelled by the film at any given instance of time, the instantaneous velocity and acceleration were calculated using a MATLAB^®^ (v. R2015 a) subroutine as a ratio of the distance travelled by the film and the corresponding time lapse. A similar approach was used to obtain the average velocity of the liquid meniscus.

## Conclusion

The enhanced wetting of a thin ferrofluidic film subjected to a non-uniform, axisymmetric magnetic field at different field intensities was studied. Image analyzing interferometry was used to explore the dynamics of the liquid film. The results indicate that the presence of a magnetic field could significantly alter the wetting dynamics of the film. The net advancement of the film, almost quintupled (from 48 μm to 206 μm) when the net magnetic force per unit volume, was varied from 20 N m^−3^ to 147 N m^−3^. The variation in the axial pull of the magnet across the two ends (capillary and adsorbed thin film region) of the film, resulted in a transient rise in the film curvature near the contact line region, followed by the advancement of the entire film with an increase in the adsorbed film thickness and a more gradual change in curvature. A theoretical model was proposed, based on a control volume approach, with a quasi-steady state assumption, taking into account the effect of the magnetic body force on the interfacial phenomenon, near the contact line. The model was further used to compute the velocity of the film at various field intensities. The evaluated velocities of the film for different field intensities corroborate the experimental observations.

## Additional Information

**How to cite this article:** Tenneti, S. *et al*. Magnetowetting of Ferrofluidic Thin Liquid Films. *Sci. Rep.* **7**, 44738; doi: 10.1038/srep44738 (2017).

**Publisher's note:** Springer Nature remains neutral with regard to jurisdictional claims in published maps and institutional affiliations.

## Figures and Tables

**Table 1:** Terminology associated with the relative distance of the magnet.

Terminology	Relative vertical position of the system from the magnet (cm), ∆Y
Set 1	4.8
Set 2	4.3
Set 3	3.7
Set 4	3.6
Set 5	3.4

**Table 2:** Variation in adsorbed thin film thickness with time and magnetic field intensity.

Set	Adsorbed thin film thickness (nm)		
	Initial	Peak	Final
1	30	22	32
2	28	22	30
3	18	16	21
4	17	13	20
5	17	12	19

**Table 3:** Net displacement of the TLF subjected to magnetic force.

Relative Axial Position of the magnet Δ*Y*, (cm)	Net Magnetic Force β (N m^−3^)	Net Displacement of the TLF from its initial position Δ*X*, (μm)
4.8	20	48
4.3	36	56
3.7	101	181
3.6	124	191
3.4	148	206
